# Artificial Intelligence for Caries Detection: Value of Data and Information

**DOI:** 10.1177/00220345221113756

**Published:** 2022-08-22

**Authors:** F. Schwendicke, J. Cejudo Grano de Oro, A. Garcia Cantu, H. Meyer-Lueckel, A. Chaurasia, J. Krois

**Affiliations:** 1 Department of Oral Diagnostics, Digital Health and Health Services Research, Charité–Universitätsmedizin Berlin, Berlin, Germany; 2 Department of Restorative, Preventive and Pediatric Dentistry, zmk bern, University of Bern, Bern, Switzerland; 3 Department of Oral Medicine and Radiology, King George’s Medical University, Lucknow, India

**Keywords:** AI, caries detection/diagnosis/prevention, computer simulation, dental informatics, economic evaluation, radiology

## Abstract

If increasing practitioners’ diagnostic accuracy, medical artificial intelligence (AI) may lead to better treatment decisions at lower costs, while uncertainty remains around the resulting cost-effectiveness. In the present study, we assessed how enlarging the data set used for training an AI for caries detection on bitewings affects cost-effectiveness and also determined the value of information by reducing the uncertainty around other input parameters (namely, the costs of AI and the population’s caries risk profile). We employed a convolutional neural network and trained it on 10%, 25%, 50%, or 100% of a labeled data set containing 29,011 teeth without and 19,760 teeth with caries lesions stemming from bitewing radiographs. We employed an established health economic modeling and analytical framework to quantify cost-effectiveness and value of information. We adopted a mixed public–private payer perspective in German health care; the health outcome was tooth retention years. A Markov model, allowing to follow posterior teeth over the lifetime of an initially 12-y-old individual, and Monte Carlo microsimulations were employed. With an increasing amount of data used to train the AI sensitivity and specificity increased nonlinearly, increasing the data set from 10% to 25% had the largest impact on accuracy and, consequently, cost-effectiveness. In the base-case scenario, AI was more effective (tooth retention for a mean [2.5%–97.5%] 62.8 [59.2–65.5] y) and less costly (378 [284–499] euros) than dentists without AI (60.4 [55.8–64.4] y; 419 [270–593] euros), with considerable uncertainty. The economic value of reducing the uncertainty around AI’s accuracy or costs was limited, while information on the population’s risk profile was more relevant. When developing dental AI, informed choices about the data set size may be recommended, and research toward individualized application of AI for caries detection seems warranted to optimize cost-effectiveness.

## Introduction

Artificial intelligence (AI) and applications from the subfield of deep learning have rapidly entered the medical arena. Especially image analysis using convolutional neural networks (CNNs) was shown to have the potential for increasing practitioners’ reliability and accuracy. CNNs learn the statistical patterns inherent in imagery by repeatedly digesting pairs of images and image labels (e.g., “this image contains a certain pathology”), with labels usually provided by medical experts, and are eventually able to assess unseen data (LeCun et al. 2015). For detecting caries lesions, we found a CNN to yield diagnostic accuracies superior to individual dentists in a diagnostic accuracy study (Cantu et al. 2020) and confirmed this in a randomized controlled trial (Mertens et al. 2021).

The detection of a pathology like a caries lesion itself does not transport any tangible value to patients or the health care system. Instead, health benefits (and further costs) emanate from the subsequent (correctly or incorrectly assigned) treatment. For caries detection on radiographs, a CNN has been found cost-effective in a modeling study, where a Markov model was used to follow detected (or nondetected) and treated (or untreated) lesions over the patients’ lifetime (Schwendicke et al. 2020). However, we also demonstrated the uncertainty involved in this cost-effectiveness.

Quantifying uncertainty is relevant for decision makers: clinicians want to know the risks of falsely relying on one instead of the other possible treatment option, in our case, using AI versus not using AI for caries detection. Health care payers may want to assess the chances of saving money when incentivizing one option over the other. Researchers desire to understand the required efforts to reduce this uncertainty. Eventually, all parties are interested in the value of information (VOI).

VOI analyses quantify the costs of making the wrong decision (more money spent than required and/or lower health benefit than possible) due to uncertainty (Ford et al. 2012), that is, they translate uncertainty into monetary value and allow to quantify the value of further information to reduce this uncertainty. For AI applications, one primary source of uncertainty stems from its diagnostic performance. Further sources are uncertain costs or the risk profile of the specific target population, for example.

Increasing the amount of data an AI model is trained on tends to increase its diagnostic performance, while in parallel, one would expect this to reduce uncertainty around the performance estimate. Assuming that the performance gains and uncertainty reductions are not linear, the monetary value of increasing the data set would not be linear, too. Moreover, these effects can be assumed to be different in different risk groups or associated with other uncertain parameters (like the costs of AI, which may grow if training is more resource intense). We aimed to quantify the value of data used to train an AI for caries detection on dental radiographs and also to assess the VOI of knowing the precise costs of the AI as well as the target population’s caries risk profile.

## Methods

### Study Design

In a previous model-based cost-effectiveness evaluation (Schwendicke et al. 2020) building on a diagnostic accuracy study (Cantu et al. 2020), we showed that using a CNN to detect caries lesions on bitewing radiographs had a high chance of being cost-effective. In the present study, we trained a CNN on a data set of cropped tooth images stemming from 3,826 bitewing radiographs and employed this health economic modeling framework for the described analyses. Reporting of this study follows the Consolidated Health Economic Evaluation Reporting Standards (CHEERS) (Husereau et al. 2013).

### Data Input

To vary the amount of training data and valuate their contribution to performance gains and certainty and, indirectly, monetary benefit, we used the imagery data set employed in our previous diagnostic accuracy study where we had trained a CNN for caries detection (Cantu et al. 2020), consisting of a total of 3,686 and 140 retrospectively collected bitewings, respectively. For the present study, each image in the training data set had been cropped tooth-wise (showing 1 tooth only) by a previously developed deep learning segmentation model, yielding 29,011 tooth crops without caries lesions and 19,760 tooth crops with caries lesions, respectively. The size of the tooth determined the size of the crop. Similarly, the test data set contained 692 tooth crops without caries lesions and 401 tooth crops with caries. Data collection had been ethically approved (ethics committee of Charité Berlin, EA4/080/18). Images had been labeled by 3 expert dentists, as well as reviewed and revised by a fourth expert. The reference test was constructed by the union of all labels.

### Model Training and Testing

To assess the impact of using more training data on performance and cost-effectiveness, the number of tooth crops employed for training/validation was incrementally increased from 10% of the total data set to 25%, 50%, and 100%, respectively, resulting in 4 different models, whose classification accuracy (true and false positive or negative findings) was employed to inform the health economic model (see below). We performed 5-fold cross-validation where the validation data set was a random sample from the training data. We used the Resnet-18 architecture pretrained on the ImageNet data set as a feature extraction module and a classification head with 2 output neurons followed by the Softmax function. Further details can be found in the Appendix.

Testing was performed on the overall test data set and on subgroups of different lesion depths (E2: lesions into the inner enamel half, D1: lesions into the outer third of dentin; D2–D3: lesions into the middle or inner third of dentin, assessed by 2 examiners in agreement).

### Setting, Perspective, Population, Horizon

We adopted a mixed public–private payer perspective in German health care (see Appendix). A population of posterior permanent teeth in initially 12-y-old individuals was modeled (TreeAge Pro 2019 R1.1; TreeAge Software). The initial age determined the horizon via the remaining lifetime of the individual (see below). The horizon was not varied across simulations.

We assumed the teeth’s proximal surfaces to start the simulation at a 1) sound, 2) initially carious (E2, D1), or 3) advanced carious status (D2–D3); the prevalence for these states had been estimated before (Schwendicke, Paris, and Stolpe 2015; Schwendicke et al. 2020) and was independent from the prevalence of lesions in our image data set used for model development. Only 1 lesion per tooth was modeled.

Besides the uncertainty stemming from the performance of the model, the caries risk profile of the population the AI is applied in has been found to introduce uncertainty (Schwendicke et al. 2020). We modeled 2 populations: 1 with low risk (low prevalence of caries lesions) and 1 with high risk (high prevalence). In the base-case analysis, we did not specify in which of these the AI was employed (i.e., introduced maximum uncertainty and quantified the VOI of reducing this uncertainty). The construction of both cohorts is described in the Appendix.

### Comparators

Similar to the original health economic study (Schwendicke et al. 2020), we compared dentists’ diagnostic accuracy when using biannual visual-tactile caries detection plus radiographic caries detection on bitewings every 2 y with that of biannual visual-tactile caries detection plus CNN-based AI for radiographic caries detection. As both dentists and AI may show different accuracy depending on the lesion stage, we used lesion depth–specific accuracies for E2, D1, and D2–D3 lesions. While visual-tactile means allowed to detect advanced (D2–D3) lesions with some accuracy, leading to restorative care (Schwendicke, Paris, and Stolpe 2015), initial lesions (E2–D1) were assumed to be only detectable using radiographs. The accuracy of dentists was built on a meta-analysis (Schwendicke, Tzschoppe, and Paris 2015), while as described, the accuracy (and the associated uncertainty) of the AI emanated from training on data sets of different size, leading to different accuracy and uncertainty and, subsequently, treatment decisions and costs.

### Cost-Effectiveness Model and Assumptions

We used a Markov simulation model, modeling posterior teeth and their proximal surfaces over their lifetime. E2–D1 were assumed be detected only radiographically and managed using microinvasive care (caries infiltration) to arrest them. If undetected or unarrested, these lesions could progress to D2–D3 lesions, which would at some point be restored using a composite restoration. Restorations could fail and be replaced or repaired, and after repeated failure, a crown was to be placed, which again could fail and be replaced once. In parallel, endodontic complications could occur, which would be treated using root canal treatment, which was also assumed to fail with some chance and then required nonsurgical and eventually surgical retreatment. If no further treatment options remained, an extraction was assumed, with teeth being replaced with implant-supported single crowns. Simulation was performed in discrete annual cycles. The transition probabilities between these different health states are shown in Appendix Table 1. The model is summarized in Appendix Figure 1.

### Input Variables

Further input variables (see Appendix) were largely built on data from large cohort studies or systematic reviews and had been validated in various health economic evaluations before (Schwendicke et al. 2013; Schwendicke, Meyer-Lueckel, et al. 2014; Schwendicke, Paris, and Stolpe 2015; Schwendicke, Stolpe, et al. 2015).

### Health Outcomes, Costs, and Discounting

Our health outcome was the time a tooth was retained (in years), mainly as valid data to assign valuations to other health states of retained teeth (e.g., nonrestored, filled, crowned tooth) are not common, while increasing research in this field may allow for more detailed consideration of cost-utility (instead of only cost-effectiveness) in the future (Hettiarachchi et al. 2018). Costs were estimated using the German public and private dental fee catalogues, Bewertungsmaßstab (BEMA) and Gebührenordnung für Zahnärzte (GOZ), and included subgroups of costs for diagnostics and treatments, as well as costs covered by insurances or out-of-pocket expenses. Costs of AI were assumed to vary between 4 and 12 euros per application (see Appendix). Costs occurring over the lifetime (i.e., in the future) were discounted at 3% per annum (IQWiG 2017).

### Analytical Methods

We first performed cost-effectiveness analysis using Monte Carlo microsimulations, with 1,000 independent teeth being followed over the mean expected lifetime (which was 66 y) (statista 2022) in annual cycles. We randomly sampled transition probabilities from uniform or triangular distributions (Briggs et al. 2002), as outlined in the Appendix. The probability that a strategy was acceptable to payers at different willingness-to-pay ceiling thresholds was also explored. In addition, we performed a range of sensitivity analyses.

Cost-effectiveness analyses indicate which strategies may be most cost-effective but accept the involved uncertainties. Reducing these uncertainties could lead to health gains or cost reductions from improved resource allocation (Claxton 1999). VOI allows to assess the foregone benefits and costs emanating from imperfect information. The VOI is estimated using the net monetary benefit (NMB), calculated as

NMB=λ×Δe−Δc,

with λ denoting the ceiling threshold of willingness to pay, that is, the additional costs (c) a decision maker is willing to bear for gaining an additional unit of effectiveness (e) (Drummond et al. 2005). For our analyses, we assumed the NMB to be λ = 0 as there are no agreed-on paying thresholds defined for an additional year of tooth retention, but also as this threshold seemed justifiable from a payer’s perspective.

VOI was then estimated as NMB_perfect information_ – NMB_imperfect information_. To estimate how perfect knowledge would change the NMB, one can identify the strategy with the highest NMB at each simulation and compare the average NMB of these “ideal” strategies with the NMB under imperfect information. We estimated the VOI of having perfect information on all uncertain parameters (expected value of information, EVPI), as well as the VOI for reducing uncertainty in specific parameters (expected value of partial perfect information, EVPPI), namely, the AI’s accuracy (amount of training data), the costs of AI, and the population’s risk profile (Ford et al. 2012). The EVPI estimates the value of simultaneously eliminating all uncertainty in an analysis, while the EVPPI can assess which parameters contribute most to the overall uncertainty.

## Results

### Study Parameters and Performance of the CNN

**Figure 1.**
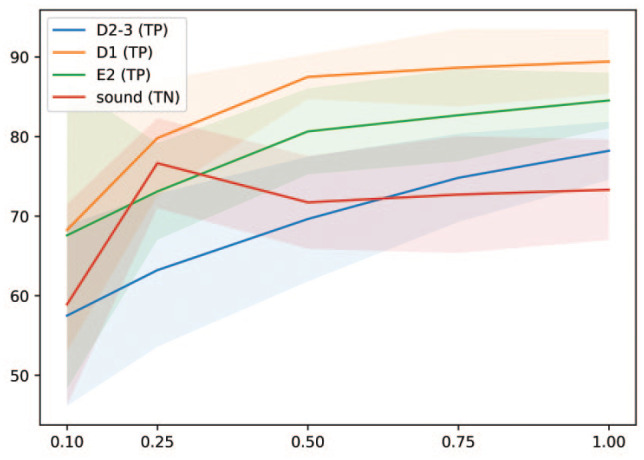
Mean true-positive (TP) and true-negative (TN) rates (in %, y-axis) for sound and carious surfaces (lines) and standard deviations (shaded areas) of artificial intelligence models trained on different proportions of the overall data set (x-axis).

The input parameters for our study are shown in Appendix Table 1. With an increasing amount of data used to train the AI, both sensitivity and specificity increased. Notably, this increase was not linear; the increase was largest when increasing the data set from 10% to 25%, and limited afterwards (see Table 2 in Appendix). The resulting percentages of true-negative (for sound surfaces) and true-positive (for carious ones) findings are displayed in Figure 1.

### Base-Case Scenario

**Figure 2.**
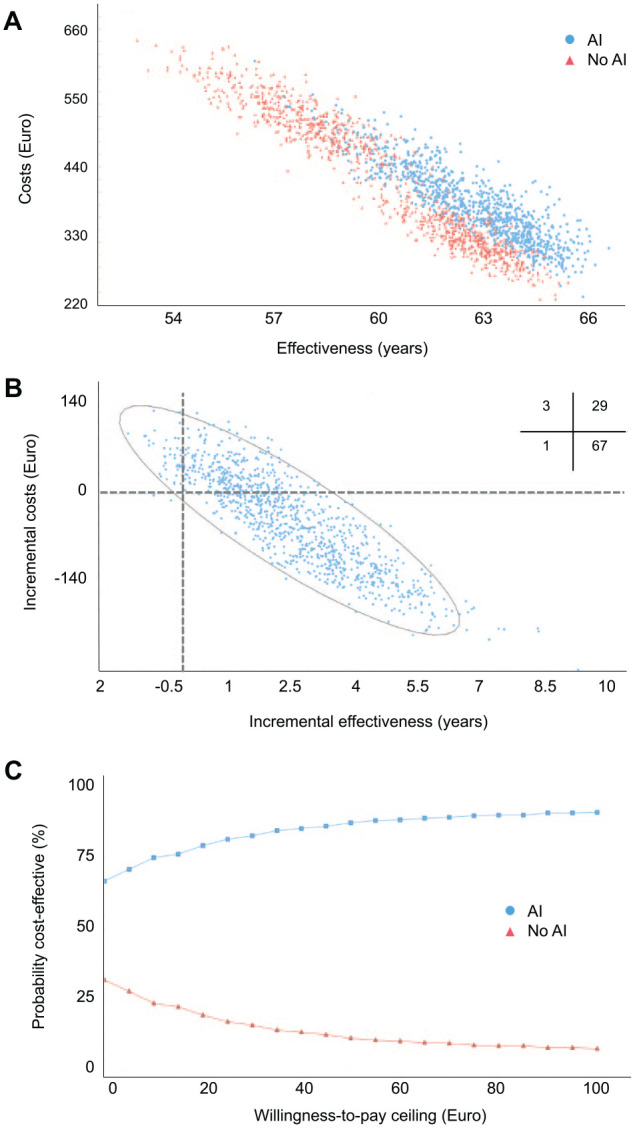
Cost-effectiveness and acceptability of the base case. (**A**) Cost-effectiveness plane. The costs and effectiveness of artificial intelligence (AI) versus no AI are plotted for 1,000 sampled individuals in each group. (**B**) Incremental cost-effectiveness. Incremental costs and effectiveness of AI compared with no AI are plotted. Quadrants indicate comparative cost-effectiveness (e.g., lower right: lower costs and higher effectiveness). Inserted cross-tabulation: Percentage of samples lying in different quadrants. (**C**) Cost-effectiveness acceptability. We plotted the probability of comparators being acceptable in terms of their cost-effectiveness depending on the willingness-to-pay threshold of a payer. The range of willingness to pay was expanded from 0 to 100 euros and did not considerably change beyond this threshold.

In the base-case scenario (uncertain accuracy of the AI, uncertain risk profile of the population, uncertain costs of AI), AI was more effective (tooth retention for a mean [2.5%–97.5%] 62.8 [59.2–65.5] y) and less costly (378 [284–499] euros) than dentists without AI (60.4 [55.8–64.4] y; 419 [270–593] euros). Figure 2 shows the cost-effectiveness plane (Fig. 2A), with AI being more effective and less costly in most simulations. This was also reflected in the incremental cost-effectiveness plane (Fig. 2B). The high cost-effectiveness acceptability was found regardless of a payer’s willingness to pay exceeding (Fig. 2C).

### Sensitivity Analyses

A range of sensitivity analyses was performed (Table 1). In low-risk populations, the cost-effectiveness of AI was lower compared with the base case (and vice versa for high-risk populations). The amount of data used for training showed a relevant effect on costs; in low-risk populations, AI was more effective but also more costly when only 10% or 25% of the data were used for training, while if more data were used for training, it was both more effective and less costly. In high-risk populations, AI was more effective and less costly regardless of the amount of data. The impact of varying the costs of AI was limited. Discounting at different rates changed the overall costs but did not change the ranking of strategies.

**Table.** Cost-Effectiveness in the Base-Case and Sensitivity Analyses.

Analysis	Dentists with AI		Dentists without AI	
	Cost (Euros)	Effectiveness (y)	Cost (Euros)	Effectiveness (y)
Base case (uncertain accuracies, uncertain AI costs, uncertain risk)	378 (284–499)	62.8 (59.2–65.5)	419 (270–593)	60.4 (55.8–64.4)
10% training data, low risk, AI costs 8 euros	379 (309–456)	63.8 (61.5–65.8)	326 (260–392)	62.4 (60.0–64.4)
25% training data, low risk, AI costs 8 euros	333 (261–410)	63.8 (60.9–65.6)	326 (260–392)	62.4 (60.0–64.4)
50% training data, low risk, AI costs 8 euros	332 (261–413)	64.1 (61.7–65.9)	326 (260–392)	62.4 (60.0–64.4)
100% training data, low risk, AI costs 8 euros	323 (250–391)	64.1 (62.1–65.7)	326 (260–392)	62.4 (60.0–64.4)
10% training data, high risk, AI costs 8 euros	451 (370–550)	61.0 (58.1–63.8)	514 (437–609)	57.9 (54.5–60.9)
25% training data, high risk, AI costs 8 euros	425 (353–506)	61.1 (58.4–63.8)	514 (437–609)	57.9 (54.5–60.9)
50% training data, high risk, AI costs 8 euros	404 (329–483)	61.8 (59.1–64.1)	514 (437–609)	57.9 (54.5–60.9)
100% training data, high risk, AI costs 8 euros	392 (318–470)	61.9 (59.7–63.9)	514 (437–609)	57.9 (54.5–60.9)
Low costs for AI (4.00 euros/analysis)	371 (275–488)	62.8 (59.2–65.5)	419 (270–593)	60.1 (55.1–64.2)
High costs for AI (12.00 euros/analysis)	392 (284–492)	62.8 (59.2–65.5)	419 (270–593)	60.1 (55.1–64.2)
Discounting rate 1%	630 (454–856)	62.8 (59.2–65.5)	745 (468–1,050)	60.4 (55.8–64.4)
Discounting rate 5%	260 (195–333)	62.8 (59.2–65.5)	270 (177–373)	60.4 (55.8–64.4)

Mean and 2.5% to 97.5% percentiles are shown. The rationale behind modeling a lower and upper bound of artificial intelligence (AI) costs of 4.00 and 12.00 euros is provided in the Appendix. The range of discounting rates follows recommendations for cost-effectiveness studies in our setting (IQWiG 2017).

### Value of Information

**Figure 3.**
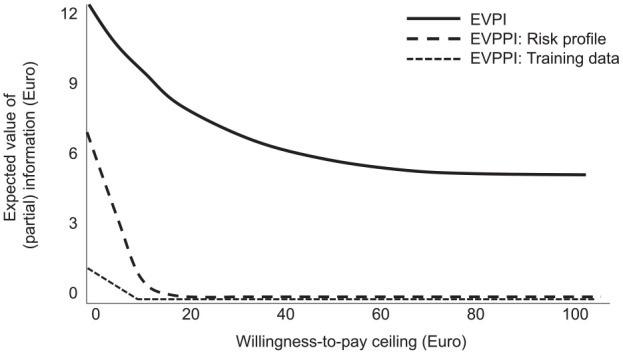
Value-of-information analysis. The overall expected value of perfect information (EVPI) and the expected value of partial perfect information (EVPPI) for specific parameters were plotted against the willingness-to-pay threshold of a payer. The EVPI and EVPPI indicate the monetary value of being able to make better decisions (avoid more costly or less effective decisions) based on better overall or partial information. EVPPI was estimated for the available data for training the artificial intelligence (AI) (affecting accuracy and its uncertainty), the risk profile (caries prevalence) of the population of interest, and the costs of AI (4–12 euros per application). For the latter, the EVPPI was 0 euros regardless of the threshold and hence not shown. The range of willingness to pay was expanded from 0 to 100 euros.

The EVPI and the EVPPI at different willingness-to-pay thresholds of a payer are shown in Figure 3. Both EVPI and EVPPI decreased with increasing willingness to pay. The EVPI at a threshold of 0 euros was 12.40 euros and decreased to a lower plateau of 5.60 euros at a higher willingness to pay. The EVPPI of training the AI with more data (affecting performance and uncertainty) was 0.87 euros at a threshold of 0 euros and flattened out to 0 euros; that of the risk profile (caries prevalence) of the population was 6.61 euros at a threshold of 0 euros and also decreased toward 0 euros at higher willingness to pay. The EVPPI of the costs of AI was 0 euros regardless of the threshold (and is hence not shown in Fig. 3).

## Discussion

While studies on the accuracy of AI applications for medical purposes are widespread, there are only few health economic evaluations of medical AI, and most of these suffer from methodological shortcomings (Wolff et al. 2020). In oral and dental research, a similar increase in studies on AI is notable, while assessments of the value of AI for dental patients, providers, or payers are scarce. Cost-effectiveness models allow to determine the potential long-term health effects and resulting costs and thereby translate accuracy into tangible value.

The present study assessed the value of enlarging the training data set used for developing an AI and, indirectly, the resulting accuracy gains (which may be nonlinear and also differ for sensitivity and specificity or different lesion stages) and uncertainty reductions. We further assess the value of knowing the costs of the AI and the population’s risk profile. In high-risk (high-prevalence) populations, even moderate sensitivity gains of AI may lead to considerable cost-effectiveness, while in low-risk populations, false-positive detections (i.e., specificity) will be more relevant.

In the present study, we showed that the benefit of more training data was not linearly increasing but saturated after limited increases in data and that in certain (high-risk) populations, AI was also cost-effective when only minimal amounts of data were used for training. It is recommendable that instead of increasing data sets on a noninformed (random) basis, researchers should identify data points that contribute to the heterogeneity of the data set and thereby increase accuracy and generalizability more efficiently. Moreover, it needs highlighting that as expected, gains in sensitivity and specificity were not identical and were further lesion stage specific, all of which had a joint impact on cost-effectiveness.

We further explored the value of reducing these (and other) uncertainties in our analysis. At 12.40 euros per individual, however, the monetary impact of eliminating all parameter uncertainty (EVPI) was limited compared with the observed lifetime costs. Moreover, and against our expectations, we showed that the value of knowing what accuracy gains are generated by which training data set size was small and that uncertainty around the costs of AI was also irrelevant. Instead, the population’s risk profile and a range of other joint uncertainties (which we did not explore in detail) were relevant. Identifying the economic value of increasing information on specific parameters helps to make informed decisions about research and development: for instance, knowledge on the caries prevalence in a specific patient pool or patients’ risk profile (e.g., by using caries risk assessment) may support a more targeted decision toward using AI or not and thereby optimize the cost-effectiveness of AI.

This study has a number of strengths and limitations. First, and as a strength, this is the first study assessing the value of training data for dental AI applications and generally one of few VOI analyses in dentistry. Our study can inform researchers, funding agencies, and developers of AI toward which uncertainties have more or less impact on health and costs. Second, the employed Markov model and the analytic framework have been validated before; they allow to extrapolate accuracy data into long-term health and economic outcomes. Third, and as a limitation, our analysis was setting specific, and so will be our results to some degree. Notably, cost estimation using German fee items has been found to closely reflect the true treatment costs and to yield estimates comparable with those from other health care settings (Schwendicke, Graetz, et al. 2014; Schwendicke et al. 2018). Fourth, construction of the reference test for training and testing the model was performed as described, with the chosen strategy being one (albeit frequently chosen) option among others. Also, we assumed early lesions to be detected radiographically, not visually, while a number of studies found visual assessment to have moderate sensitivity for detecting early proximal lesions, too (Foros et al. 2021). Fifth, the accuracy values assumed in our control group (dentists without AI) stemmed from a systematic review that also confirmed that many of the included diagnostic accuracy studies suffered from bias and limited applicability. Notably, we have investigated the impact of different accuracy values in the control group in a previous cost-effectiveness study (Schwendicke 2020) and did not find the introduced variances in accuracy to change our conclusion. Moreover, we have assessed the cost-effectiveness of AI for this purpose not only against systematically reviewed and synthesized data but also recent data from a prospective controlled trial (Schwendicke et al. 2022). In the present study, our focus was not on the comparative cost-effectiveness but the uncertainty around it. Last, our simulation simplified decision making in practice; dentists may deviate from AI detections and apply a range of therapies beyond those assumed in our study. The latter point is relevant, as the assigned treatment has been shown to affect cost-effectiveness (Schwendicke et al. 2020).

In conclusion, and within the limitations of this study, increasing the amount of data for training an AI to detect caries lesions on bitewings improved cost-effectiveness. Notably, limited increases in data led to significant increases in cost-effectiveness, and enlarging the data set even further was of limited benefit. There was considerable uncertainty around the cost-effectiveness. The economic value of reducing this uncertainty, specifically around the AI’s accuracy or costs, was limited, though. Instead, the risk profile of the population of interest was more important. When developing dental AI, informed choices about the data set size may be recommended, and research toward individualized application of AI for caries detection seems warranted to optimize cost-effectiveness.

## Supplemental Material

sj-docx-1-jdr-10.1177_00220345221113756 – Supplemental material for Artificial Intelligence for Caries Detection: Value of Data and Information Click here for additional data file. Supplemental material, sj-docx-1-jdr-10.1177_00220345221113756 for Artificial Intelligence for Caries Detection: Value of Data and Information by F. Schwendicke, J. Cejudo Grano de Oro, A. Garcia Cantu, H. Meyer-Lueckel, A. Chaurasia and J. Krois in Journal of Dental Research
