# Supplementary material for: Artificial Intelligence for Caries Detection: Value of Data and Information
Source: J Dent Res. 2022 Aug 22;101(11):1350–6. doi: 10.1177/00220345221113756 (PMC9516598; doi:10.1177/00220345221113756)
Supplement: sj-docx-1-jdr-10.1177_00220345221113756 – Supplemental material for Artificial Intelligence for Caries Detection: Value of Data and Information [file sj-docx-1-jdr-10.1177_00220345221113756.docx]

**Appendix**

*Image dataset and model training*

Bitewings were collected between 2016 and 2018. Bitewings of permanent teeth, with at least the crowns of one dental arch being detectable, were included. There were 52% male and 48% female patients; the mean (SD, min-max) age was 36.5 (14.3, 14-89) years.

For yielding models on differently sized training datasets, we stepwise increased the training dataset. The test dataset was kept fixed across experiments, and therefore its size didn't change. This ensured the evaluation as consistent across experiments since all the models were evaluated using the same dataset. For each trained model accuracy was calculated on the test set in a typical manner, as the ratio between the correctly classified images and the total number of datapoints.

The models were trained for a maximum of 50 epochs, monitoring the performance on the validation set and using early stopping with a patience parameter of 5 epochs to avoid overfitting. We used a batch size of 64 images and the Adam optimizer with a learning rate of 1e-4. Due to the slight imbalance of the dataset, we used a weighted cross-entropy loss function with weights inversely proportional to the frequency of each category. The images were resized into 256x256x3 tensors and were normalized with the mean and standard deviation of the ImageNet dataset. We used vertical and horizontal flipping as image augmentations for training. The models were trained on a NVIDIA Quadro RTX 6000 graphics card (NVIDIA, Santa Clara, CA, USA) using the deep learning framework PyTorch.

*German healthcare*

In Germany, the medical insurance is two-tiered, with most individuals (>87%) being publicly insured (statutory insurance) and only a minority being privately insured. For members of the statutory insurance, nearly all dental procedures are fully covered, while only few need to be partially or fully paid out-of-pocket or by private (additional) insurances.

*Prevalence*

As outlined elsewhere (Schwendicke et al. 2015a), simulations were performed for two populations – one with low caries prevalence, and one for a population with high prevalence. Prevalence was estimated from the number of proximal surfaces with enamel and dentin caries lesions reported for 12-year-olds from Sweden under a preventive dental regime with low caries risk, assuming only one surface per tooth to bear a lesion and 16 posterior teeth being at risk (Mejàre et al. 2004). We assumed that 87% of dentinal lesions would be extended into the outer, not inner dentin at age 12. Prevalence was adjusted for populations with high risk (Micheelis and Schiffner 2006). Note that this does not necessarily reflect the “true” prevalence, but allows to simulate possible ranges of caries prevalence.

Note that the prevalence of caries lesions in the sample used to train our model was higher, which benefits training and increases model accuracy (having a balanced or nearly balanced dataset is helpful when training deep learning models, and strategies to re-balance imbalanced datasets are often employed (e.g. upsampling etc). Also note that the prevalence in the training dataset determines the effectiveness of learning, while it was relevant to use realistic prevalences during cost-effectiveness and value of information modeling. This was ensured in the present study by constructing “artificial” but representative, simulated cohorts, as laid out above.

*Cost estimation*

German dentists use fee items to claim for reimbursement for dental treatments. For most dental procedures and patients, items will be drawn from the public catalogue BEMA. For few treatments (composite restorations in posterior teeth, implants and ISC), fees are usually derived from the private catalogue; publicly insured patients pay the additional costs ouf-of-pocket or via additional private insurers For GOZ, factoring of the chargeable item points is common to determine the fees of private treatment in Germany; we used the standard multiplication factor (2.3). Using fee items allowed us to estimate costs occurring to payers, which was in line with our study perspective (Schwendicke et al. 2013a).

BEMA defines fee items within the public insurance, which covers 87% of insured Germans (GKV-Spitzenverband 2013), with only few treatments not being fully covered or reimbursed. For these items, calculation was based on GOZ or “analogue-items”. BEMA points vary between federal states, insurers and treatment groups. For our cost calculation we used mean state points for the biggest insurer (AOK) from one federal state, Bavaria. For GOZ, point values were applied, with 0.0562421 Euro/point. Factoring of GOZ item-points was usually performed via the standard multiplication factor (×2.3). Certain positions (mainly radiographic assessments) are further coded in GOÄ (Gebührenordnung für Ärzte), with 9 GOÄ points equaling 1 BEMA point. Laboratory and material costs were estimated based on Laboratory Fee Catalogues (BEL II/BEB). Costs for BELII/BEB have been transformed into monetary values for the following tables. Items were restricted in number and character to reflect cost limitations. Total costs per course of treatment were calculated based on the quantification (q) of itemized costs (c), i.e. c_1_×q_1_ + c_2_×q_2_ etc., and calculated in Euro. Details can be found elsewhere (Schwendicke et al. 2020).

*Costs of AI*

We assumed the following costs to have occurred for establishing the AI intervention:

- Data generation and labeling: depending on the proportion of the overall training dataset being employed for training, we assumed up to 3,686 images being assessed by 4 annotators. We assumed each annotation to require 5 min per image. At an average salary incl tax and social security contributions the costs were approx. 39,- Euro/h (TV-Ä Charité); in total 47.918 Euro for the complete dataset.
- Training of the AI model, approx. 4 months data science costs, at monthly costs for 5.750,- Euro (TV-E13), in total 23.000 Euro
- Software engineering costs for the intervention, approx. 12 months full time at 5.750,- Euro (TV-E13), in total 69.000 Euro
- Regulatory process incl. user tests, clinical evaluation, technical documentation and quality management system (might be available already, though), approx. 100,000 Euro
- Total development costs: 239,918 Euro. These were assumed to be distributed over the first 100,000 use cases, i.e. analyses, i.e. 2.40 Euro per image. Notably, the data generation and labeling costs were 19.97% of the overall costs in case the full dataset, i.e. 0.48 Euro per use. Costs for data generation and labeling were reduced accordingly to 1.997% (0.05 Euro), 4.993% (0.12 Euro), and 9.995% (0.24 Euro) for the 10%, 25% and 50% datasets, respectively.

We further assumed costs per goods sold (COGS) to occur as follows:

- Cloud infrastructure. According to our simulations, costs of 1-3 Euro per image analysis occur depending on the load and the accepted waiting time for each dentist.
- Support etc. including possible step-by-step instructions; we assumed one monthly email and one monthly phone contact, each consuming 10 min of a support employee’s time, i.e. 39,- Euro per hour (TV-E13), in total 13 Euro/ month. Assuming each dentist to use the intervention for each bitewing, at an estimated number of 20 bitewings per month, this summed up to 0.65 Euro per month.
- The total COGS hence summed up to 1.65-3.65 Euro/image.

Total costs per image were hence assumed to be 4.05 Euro to 6.05 Euro per analysis. We further assumed any provider of such a tool would also need to generate additional revenue for management, sales and marketing, development and profit (overhead), and assumed this block to account for 55% of the abovementioned costs (i.e. 2.24-3.33 Euro). Hence, total costs were assumed to range between 6.29-9.38 Euro. Given that developmental costs would, however, be diluted with each use case exceeding 100,000 analyses, and given that the costs for revenue, sales and marketing, development and profit would also be reduced if the intervention was scaled up in its use, our cost estimate between 4-12 Euro seemed to realistically cover possible cost scenarios.

*Figures and tables*


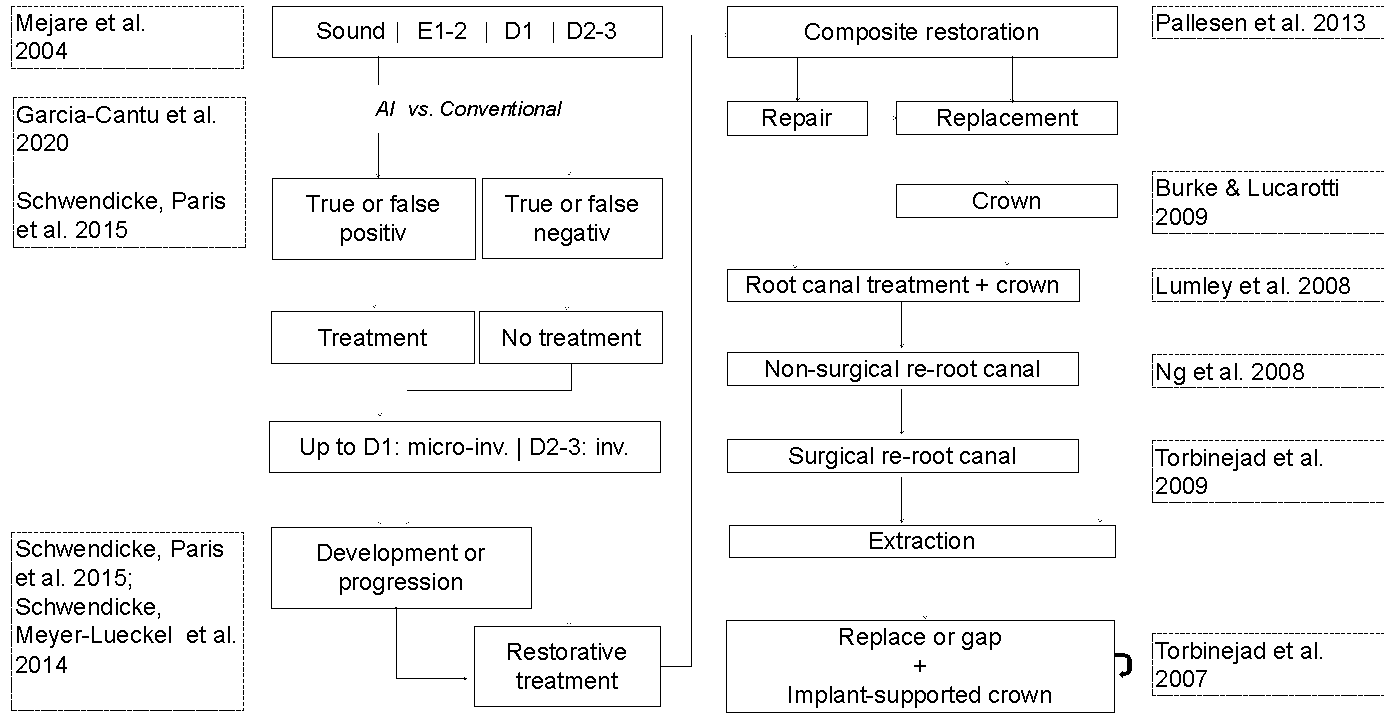


Appendix Figure S1: Input data and model. The state diagram (central parts) shows the different health states (solid boxes). Transition or allocation probabilities determined the chance of passing between them, indicated by arrows. The data sources used to simulate individuals’ flow through the model are shown in dotted boxes at the left and right; references can be found in the main text. Individuals started with teeth being either sound or showing E1/2/D1 and D2-3 lesions. Sound surfaces could be detected as such, without any subsequent treatment, or false positively detected as initial (E2/D1) caries lesions depending on the detection method. False positive detections on sound surfaces led to infiltration treatment, without any effectiveness gain, but money spent unnecessarily. Initial lesions (E2/D1) could again be detected (treated by resin infiltration) or not detected (and assumed to progress with some chance) and depending on the efficacy of resin infiltration be arrested, or progress to D2 lesions. For advanced lesions (D2) not extending into the inner third of the dentin (D3), a two-surfaced restoration was assumed to be placed. The risk of restorative complications was derived from previous studies, and if restorations failed, they were assumed to be either renewed or repaired. If failing again, the placement of a full-metal crown was assumed. Failed crowns were assumed to be replaced once, afterwards which the tooth was extracted. Extracted teeth were assumed to be replaced using implant supported crowns.

Appendix Table 1: Input parameters. Uncertainty was introduced by random sampling within distributions as indicated.

|  | | **Prevalence, accuracy, lesion development and progression** | | | | | | | | |  | | | |  | | | | |  |  |
| --- | --- | --- | --- | --- | --- | --- | --- | --- | --- | --- | --- | --- | --- | --- | --- | --- | --- | --- | --- | --- | --- |
| **Estimate** | **Source (reference)** | | | | **No lesions (sound)** | | | | **Lesions into  inner third of enamel (E2)** | | **Lesions into  outer third of dentin (D1)** | | | | | **Lesions into  middle third of dentin (D2)** | | | |  |  |
| **Prevalence** | | | |  | | | |  | | | | | |  | | | | |  |  |  |
| Low risk | (Schwendicke et al. 2015a) | | | | | 0.17 | | | | 0.14 | | 0.025 | | | | | 0.005 | | | | |
| High risk | (Schwendicke et al. 2015a) | | | | | 0.35 | | | | 2.14 × 0.14 | | 1.66 × 0.025 | | | | | 1.66 × 0.005 | | | | |
| **Sensitivity and specificity** | | | |  | | | |  | | | | | |  | | | | |  |  |  |
| Visual-tactile* | (Schwendicke et al. 2015a) | | | | | TN: 0.995 | | | | TP: 0.005 | | TP: 0.005 | | | | | TP: 0.31 | | | | |
| Radiography no AI* | (Schwendicke et al. 2015b) | | | | | TN: 0.96 | | | | TP: 0.24 | | TP: 0.36 | | | | | TP: 0.64 | | | | |
|  |  | | | | |  | | | |  | |  | | | | |  | | | | |
| **Probability of lesion development** | (Schwendicke et al. 2015a) | | | | |  | | | | p=1.26 × 0.57252 × 2.7^-0.1472 × 2α^ distribution: 1.24-1.29 | | p=1.26 × 0.0426 × 2.7^-0.0521 × 2α^  distribution: 1.24-1.29 | | | | | p=1.26 × 0.57 × 0.0426 × 2.7^-0.0521 × 2α^  distribution: 1.24-1.29 | | | | |
|  |  | | | | |  | | | |  | |  | | | | |  | | | | |
| **Probability of lesion progression** | | |  | | | |  | | | | | |  | | | | |  |  |  |  |
| Progression to |  | | | | |  | | | | D1 lesion | | D2 lesion | | | | | D3 lesion | | | | |
| If untreated | (Schwendicke et al. 2015a) | | | | |  | | | | p=2.63 (high risk) / 2.13 (low risk) × 3.0984×(2α)^-1.343^  (distribution: p × 0.87 – p × 1.13) | | p=2.63 (high risk) / 2.13 (low risk) × 161.52×(2α)^-2.078^  (distribution: p × 0.87 – p × 1.13) | | | | | p=1.32 × 161.52 ×  (2α)^-2.078^  (distribution: p × 0.87 – p × 1.13) | | | | |
| If infiltrated | (Schwendicke et al. 2015a) | | | | |  | | | | p=0.4289×(2α)^-1.391^  (distribution: p × 0.23 – p × 5.15) | | p=68.869×(2α)^-2.078^  (distribution: p × 0.23 – p × 4.17) | | | | | - | | | | |
| **Transition probabilities** | | | | |  | | | |  | |  | | | | |  | | | | |  |
| **Health state** | **Source (reference)** | | | | |  | | | | **Transition probability per cycle** | | **Transition to** | | | | | **Allocation probability** | | | | |
| Composite^1^ | (Pallesen et al.) | | | | |  | | | | 0.016 | | Composite  Crown  Repair  RCT  Extraction | | | | | 0.45  0.10  0.10  0.25  0.10 | | | | |
| Direct capping^2^ | (Schwendicke et al. 2013b) | | | | |  | | | | 0.111 | | RCT  Extraction | | | | | 0.95  0.05 | | | | |
| Crown on vital tooth^3^ | (Burke and Lucarotti 2009) | | | | |  | | | | 0.036 | | RCT  Recementation  Repair  Re- crown  Extraction | | | | | 0.25  0.15  0.10  0.40  0.10 | | | | |
| Root canal treatment | (Lumley et al. 2008) | | | | |  | | | | 0.021 | | Non-surgical re-treatment Surgical re-treatment Extraction | | | | | 0.20  0.30  0.50 | | | | |
| Crown on non-vital tooth^3^ | (Burke and Lucarotti 2009) | | | | |  | | | | 0.029 | | Recementation  Repair  Re- crown^3^  Extraction | | | | | 0.20  0.10  0.60  0.10 | | | | |
| Non-surgical root-canal treatment | (Ng et al. 2008) | | | | |  | | | | 0.085 | | Surgical re-treatment  Extraction | | | | | 0.25  0.75 | | | | |
| Surgical root-canal treatment | (Torabinejad et al. 2009) | | | | |  | | | | 0.061 | | Extraction | | | | | 1.00 | | | | |
| Implant and implant-supported crown | (Torabinejad et al. 2007) | | | | |  | | | | 0.010 | | Recementation/Refixing Re-crown  Re-implant | | | | | 0.60  0.20  0.20 | | | | |

* Ranges were modelled as outlined previously (Schwendicke et al. 2020).

^1^ Data from 15-19-year-olds. Risk of pulpal exposure during re-composite assumed to be 10%. Crowning assumed if re-restored before.

^2^ 95% of exposed pulps were treated using direct capping, 5% were assumed to receive immediate root canal treatment.

^3^ For non-vital crowned teeth, risk of endodontic complications was calculated separately (Ferrari et al. 2012).

Analyses were performed for populations with low and high caries prevalence and risks, respectively. The probabilities of lesion development and progression if untreated or infiltrated were calculated according to patient’s age (α) using hazard functions. Other transition probabilities were extracted from large cohort studies or systematic reviews; allocation probabilities were similarly derived from cohort studies or claims data analyses, or informed by clinical experience, as described in the main text.

Appendix Table 2: Mean accuracy, sensitivity and specificity of the model trained with different sample sizes

| **Training data proportion** | **Accuracy** | **Sensitivity** | **Specificity** |
| --- | --- | --- | --- |
| 0.1 | 0.601 | 0.628 | 0.585 |
| 0.25 | 0.724 | 0.658 | 0.763 |
| 0.50 | 0.732 | 0.740 | 0.726 |
| 0.75 | 0.737 | 0.744 | 0.732 |
| 1.0 | 0.746 | 0.760 | 0.737 |

**References for the appendix**

Burke FJT, Lucarotti PSK. 2009. Ten-year outcome of crowns placed within the general dental services in england and wales. Journal of dentistry. 37(1):12-24.

Ferrari M, Vichi A, Fadda GM, Cagidiaco MC, Tay FR, Breschi L, Polimeni A, Goracci C. 2012. A randomized controlled trial of endodontically treated and restored premolars. Journal of Dental Research. 91(7 suppl):S72-S78.

GKV-Spitzenverband. 2013. Official statistics january [amtliche statistik km1].

Lumley PJ, Lucarotti PSK, Burke FJT. 2008. Ten-year outcome of root fillings in the general dental services in england and wales. International Endodontic Journal. 41(7):577-585.

Mejàre I, Stenlund H, Zelezny-Holmlund C. 2004. Caries incidence and lesion progression from adolescence to young adulthood: A prospective 15-year cohort study in sweden. Caries Research. 38(2):130-141.

Micheelis W, Schiffner U. 2006. Vierte deutsche mundgesundheits-studie (dms iv). Köln Deutscher Ärzteverlag.

Ng YL, Mann V, Gulabivala K. 2008. Outcome of secondary root canal treatment: A systematic review of the literature. International Endodontic Journal. 41(12):1026-1046.

Pallesen U, van Dijken JWV, Halken J, Hallonsten A-L, Höigaard R. 2013. Longevity of posterior resin composite restorations in permanent teeth in public dental health service: A prospective 8 years follow up. Journal of Dentistry. 41(4):297-306.

Schwendicke F, Paris S, Stolpe M. 2015a. Detection and treatment of proximal caries lesions: Milieu-specific cost-effectiveness analysis. Journal of dentistry. 43(6):647-655.

Schwendicke F, Rossi JG, Göstemeyer G, Elhennawy K, Cantu AG, Gaudin R, Chaurasia A, Gehrung S, Krois J. 2020. Cost-effectiveness of artificial intelligence for proximal caries detection. Journal of dental research.22034520972335.

Schwendicke F, Stolpe M, Meyer-Lückel H, Paris S, Dörfer C. 2013a. Cost-effectiveness of one- and two-step incomplete and complete excavation. Journal of Dental Research. 92(10):880 - 887.

Schwendicke F, Stolpe M, Meyer-Lueckel H, Paris S, Dörfer CE. 2013b. Cost-effectiveness of one- and two-step incomplete and complete excavations. Journal of Dental Research. 90(10):880-887.

Schwendicke F, Tzschoppe M, Paris S. 2015b. Radiographic caries detection: A systematic review and meta-analysis. J Dent. 43(8):924-933.

Torabinejad M, Anderson P, Bader J, Brown LJ, Chen LH, Goodacre CJ, Kattadiyil MT, Kutsenko D, Lozada J, Patel R et al. 2007. Outcomes of root canal treatment and restoration, implant-supported single crowns, fixed partial dentures, and extraction without replacement: A systematic review. The Journal of prosthetic dentistry. 98(4):285-311.

Torabinejad M, Corr R, Handysides R, Shabahang S. 2009. Outcomes of nonsurgical retreatment and endodontic surgery: A systematic review. Journal of Endodontics. 35(7):930-937.
